# Unraveling Molecular Differences of Gastric Cancer by Label-Free Quantitative Proteomics Analysis

**DOI:** 10.3390/ijms17010069

**Published:** 2016-01-21

**Authors:** Peng Dai, Qin Wang, Weihua Wang, Ruirui Jing, Wei Wang, Fengqin Wang, Kazem M. Azadzoi, Jing-Hua Yang, Zhen Yan

**Affiliations:** 1 State Key Laboratory of Cancer Biology, Department of Pharmacogenomics, School of Pharmacy, Fourth Military Medical University, Xi’an 710032, China; rarfen@163.com (P.D.); maomao_snnu@163.com (Q.W.); weihuawang@email.arizona.edu (Wh.W.); wangwei_fmmu@163.com (W.W.); 2 The Cancer Research Center, School of Medicine, Shandong University, Jinan 250012, China; jingruirui223@163.com (R.J.); 15554139558@163.com (F.W.); 3 Departments of Surgery and Urology, Boston Veterans Affairs Healthcare System, Boston University School of Medicine, Boston, MA 02130, USA; kazadzoi@bu.edu

**Keywords:** gastric cancer, LC-MS/MS, Label-free quantitative proteomics, heterogeneous nuclear ribonucleoprotein, Y-box binding protein 1

## Abstract

Gastric cancer (GC) has significant morbidity and mortality worldwide and especially in China. Its molecular pathogenesis has not been thoroughly elaborated. The acknowledged biomarkers for diagnosis, prognosis, recurrence monitoring and treatment are lacking. Proteins from matched pairs of human GC and adjacent tissues were analyzed by a coupled label-free Mass Spectrometry (MS) approach, followed by functional annotation with software analysis. Nano-LC-MS/MS, quantitative real-time polymerase chain reaction (qRT-PCR), western blot and immunohistochemistry were used to validate dysregulated proteins. One hundred forty-six dysregulated proteins with more than twofold expressions were quantified, 22 of which were first reported to be relevant with GC. Most of them were involved in cancers and gastrointestinal disease. The expression of a panel of four upregulated nucleic acid binding proteins, heterogeneous nuclear ribonucleoprotein hnRNPA2B1, hnRNPD, hnRNPL and Y-box binding protein 1 (YBX-1) were validated by Nano-LC-MS/MS, qRT-PCR, western blot and immunohistochemistry assays in ten GC patients’ tissues. They were located in the keynotes of a predicted interaction network and might play important roles in abnormal cell growth. The label-free quantitative proteomic approach provides a deeper understanding and novel insight into GC-related molecular changes and possible mechanisms. It also provides some potential biomarkers for clinical diagnosis.

## 1. Introduction

Gastric cancer (GC) has long been recognized as among the world’s major malignancies, ranked fifth in incidence and third in mortality since 2012 [1,2,3]. In China, approximately 405,000 new cases and 325,000 deaths from GC have been reported, making it the second most prevalent disease and the third in cancer-related deaths [2].

GC is a complex and multi-factorial process disease, resulting from the interaction between genetic and environmental factors that potentially deregulate cell oncogenic signaling pathways to promote GC development [4,5]. Early onset GC is difficult to diagnose due to the histological and genetic heterogeneity of the disease. Most patients are asymptomatic in the initial stages, making it difficult to control the malignancy rate through early detection and motivational therapy. GC patients are often diagnosed after the disease has progressed to the advanced stage where the long term outlook is very poor (5-year survival rate of 10%–20%) [3,6,7]. Common methods for detecting GC are endoscopy and biopsy, which are both time-consuming and invasive, and can only identify the disease at a relatively late stage [8]. Moreover, these traditional detection methods are costly and not suitable for large-scale preventive screening [9]. Treatment of GC includes surgery, chemotherapy and radiotherapy, however the effects are limited. In recent years, the development of molecular targeted therapy has led to a revolutionary breakthrough in clinical therapy and become the hope of cancer treatment [6]. Several molecular signaling pathways related to cell proliferation, invasion, angiogenesis and metastasis have been identified and evaluated as candidates for targeted treatment. Despite promising pre-clinical data, the majority of targeted agents failed to improve outcome, and therapeutic advances in GC lag well behind other challenging organ malignancies [6]. Effective targeted therapy depends on identifying cancer driving molecules and key signaling pathways. Thus, a better understanding of gastric carcinogenesis through proteomic and genetic studies can provide important novel insights into development and progression of GC, and can be utilized to improve early diagnostic screening and provide effective drug intervention targets [10]. However, these advancement can only be achieved through the use of new technologies and methods.

The use of omics technologies is quite suitable to characterize molecular pathogenic mechanisms and signaling networks, and identify disease biomarkers, involving multi-factor and genetic factors [11]. Proteomics can resolve molecular details of proteome variation in tissues from different human organs, increasing our knowledge about human biology and disease, reflecting more accurately on the dynamic state of biological fluid, organelle, cell, tissue, organ, system, or the whole organism, and yielding better disease markers for diagnosis and therapy monitoring [12,13]. Therefore, establishing in-depth proteomics profiles of various biospecimens obtained from cancer patients are expected to increase our understanding of tumor pathogenesis, improve therapy monitoring, and identify novel targets for cancer treatment [14]. In recent years, mass spectrometry-depend proteomic techniques, including 2D-DIGE (fluorescence 2-dimensional difference gel electrophoresis), iTRAQ (isobaric tags for relative and absolute quantification), ICAT (isotope-coded affinity tag), SILAC (stable isotope labeling with amino in cell culture), AQUA (absolute quantification) and label-free quantitative proteomics have become more advanced and are now being applied to quantitatively analyze proteins differences in various disease [12].

Label-free quantitative proteomics has broad superiorities in discovery of biomarkers or drug targets and has gained more popularity in recent years [15,16]. There is no need for any isotopic or chemical labeling, so it does not require extra experimental steps and the limitation caused by the labeling can be omitted [15,17]. Label-free approach has the largest dynamic range and the highest proteome coverage for identification. Comparative quantification of label-free approach can be performed across many complex samples simultaneously, especially ideal for investigating proteins relatively low in abundance [16]. Since the introduction of label-free proteomics, a broad variety of studies interested in understanding molecular mechanisms and pathways, and discovery of biomarkers or drug targets of diverse diseases have been performed [16,18].

Although many studies in the field of proteomics have been used to screen dysregulated proteins and to identify potential biomarkers or drug targets in various complex samples of GC [19,20], label-free proteomics has been applied to few GC cases. In this study, our objective was to investigate GC-related differential proteins in the proteome of patients’ tissues to find any potential molecular and signaling networks, to reveal potential carcinogenic mechanisms, and to evaluate any specific biomarkers for GC diagnosis and treatment by a coupled label-free MS approach.

## 2. Results

### 2.1. Overall Protein Changes Identified by Label-Free Quantitative Strategy

**Figure 1:**
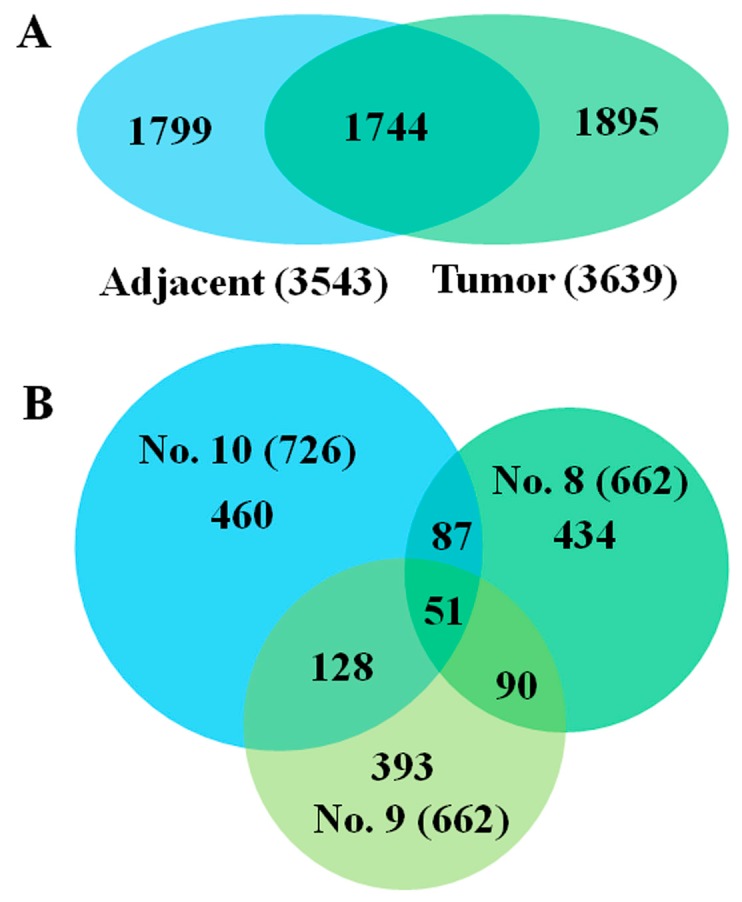
Venn diagrams of total identified proteins and dysregulated proteins. (**A**) Total proteins identified from three cases in tumor or adjacent tissues respectively. One thousand seven hundred forty-four proteins identified appear in both tumor and adjacent tissues; (**B**) The number of proteins with more than twofold differential expression in three cases, respectively, and the number of proteins shared in two or three cases.

To quantitatively compare GC tissue proteome, proteins were extracted from surgically resected fresh cancer and adjacent tissues of three patients, randomly selected from ten patient samples. After in-solution tryptic digestion, each of three samples were run in triplicate by LC-MS/MS, and a total of 3639 and 3543 proteins were identified by Proteome Discoverer 1.4 in three pairs of GC and adjacent tissues, respectively (Figure 1A and Table S1, Supplementary Material). After evaluating MS data quality, all three samples were analyzed with Progenesis LC-MS software, using an algorithm based on the pair-wise features detection at LC-MS level. 726, 662 and 662 dysregulated proteins (≥2-fold) were quantified in three GC patients (Figure 1B and Table S1, Supplementary Material), while 146 dysregulated proteins were reliably quantified after limited selection in these proteins. Of 146 proteins, 81 proteins were downregulated, 65 proteins were upregulated (adjacent/tumor ratio ≥2-fold or ≤0.5-fold, *p*-value < 0.05) and 22 proteins were first found to be related with GC (Table S1, Supplementary Material).

**Figure 2:**
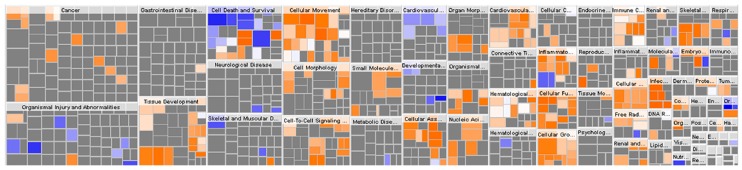
The hierarchical heatmap of 146 dysregulated proteins analyzed by Ingenuity Pathway Analysis (IPA)**.** The major boxes represent specific family or category of related functions. The smaller squares within the major boxes represent the number of proteins. Each individual square represent a specific protein. Colored squares indicate protein predicted state: increasing (orange), or decreasing (blue). Darker colors indicate higher absolute Z-scores.

### 2.2. Functional Annotation of Proteins between GC and Adjacent Tissues

To reveal any possible function of the 146 dysregulated proteins, *in silico* analysis was performed. Ingenuity Pathway Analysis (IPA) aids in the integration of complex omics data and provides insight into regulatory mechanism and biological functions based on published studies [21]. The heatmap of “disease and function” of 146 dysregulated proteins by IPA was shown in Figure 2, most of these proteins were involved in cancers (117/146, 80.14%) and gastrointestinal disease (99/146, 67.80%) (Table 1 and Table S2 (Supplementary Material)). Their main functions concern cellular growth and proliferation, nucleic acid metabolism, small molecule biochemistry, cell death and survival, cellular movement (Table 2 and Table S2 (Supplementary Material)).

**Table 1:** Dysregulated proteins and related disorders analyzed by IPA.

Disease and Disorder	No. of Molecules	*p*-Value	Protein Names
Cancer	117	2.62 × 10^−11^–2.63 × 10^−3^	ANPEP, ANXA1, ATP2A2, ATP4A, CBX3, HNRNPA2B1, HNRNPC, HNRNPL, HSP90AB1, ILF2, NPM1, RAN, SNRPF, VIM, YBX1, …
Gastrointestinal Disease	99	2.62 × 10^−11^–2.98 × 10^−3^	ANPEP, ANXA1, FN1, HNRNPA2B1, HNRNPC, HNRNPL, HPX, NPM1, RAN, SFN, SNRPF, TAGLN, VIM, WARS, YBX1, …

**Table 2:** Molecular and cellular function of dysregulated proteins analyzed by IPA.

Function	No. of Molecules	*p*-Value	Protein Names
Cellular Growth and Proliferation	78	4.95 × 10^−13^–2.42 × 10^−3^	ACAT1, HNRNPA2B1, HNRNPC, HNRNPD, HNRNPL, HNRNPR, HPX, HRG, HSP90AB1, HSPB1, LF2, NPM1, RAN, VIM, YBX1, …
Nucleic Acid Metabolism	25	7.36 × 10^−12^-1.83 × 10^−3^	ACAA2, ATP2A2, ATP4A, ATP4B, CS, CYCS, EIF4A3, HMGCL, PNP, PPA1, SET,SOD1, TYMP, VCP, VDAC1, …
Small Molecule Biochemistry	36	7.36 × 10^−12^–3.02 × 10^−3^	ANXA1, ATP2A2, ATP4A, ATP4B, CMPK1, CYCS, EIF4A3, MT-ATP6, PNP, PPA1, SET, SOD1, TYMP, VCP, VDAC1, …
Cell Death and Survival	74	1.98 × 10^−10^–2.53 × 10^−3^	ACAT1, CCT2, CFH, CP, CTNNB1, CYCS, DPYSL3, EZR, F13A1, FGG, FN1, HNRNPC, NPM1, VIM, YBX1, …
Cellular Movement	52	5.38 × 10^−9^–2.97 × 10^−3^	ACTN4, ANXA1, CNN1, CTNNB1, DPYSL3, FN1, HNRNPA2B1, HNRNPL, HRG, HSP90AB1, NPM1, SFN, VIM, WARS, YBX1, …

**Figure 3:**
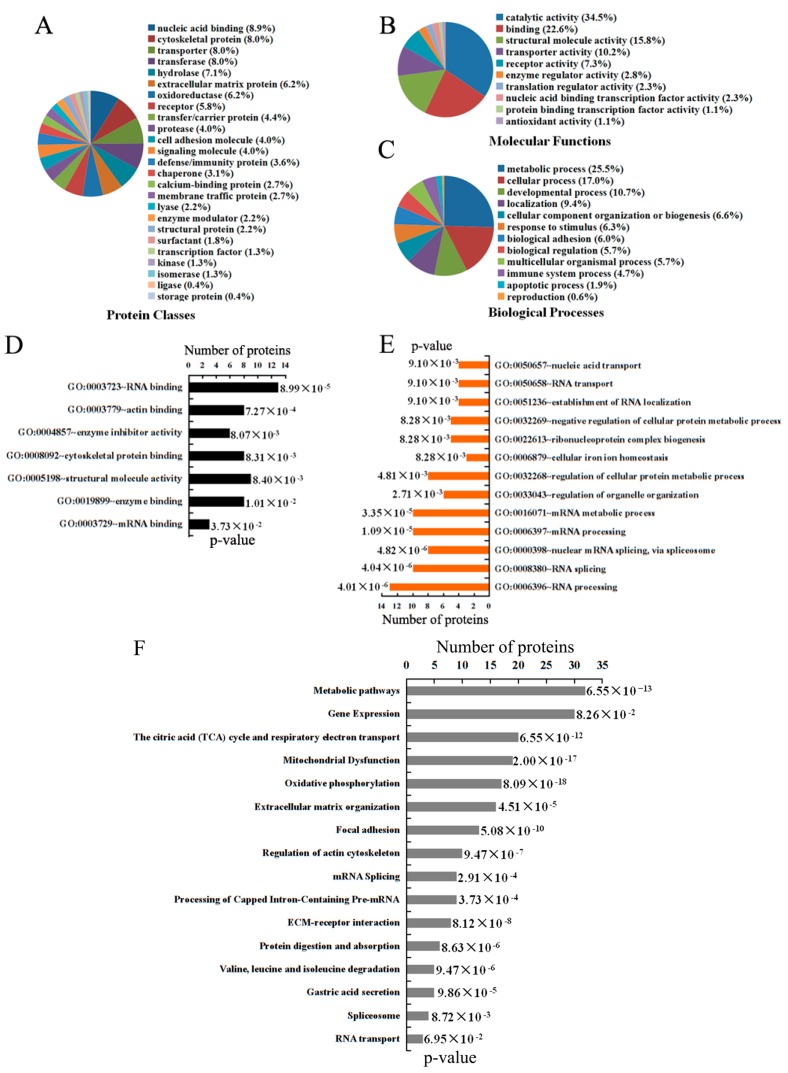
The functional annotation of dysregulated proteins was analyzed by Protein Analysis Through Evolutionary Relationships (PANTHER), Database for Annotation, Visualization and Integrated Discovery (DAVID), STRING and Reactome. (**A**) Protein Classes; (**B**) Biological Process; and (**C**) Molecular Function of 146 dysregulated proteins were summarized in a pie chart by PANTHER; (**D**) Molecular function; and (**E**) Biological process based on the 65 upregulated proteins were depicted in a bar graph by DAVID; (**F**) Pathway analysis of 146 dysregulated proteins was indicated by PANTHER, DAVID, STRING and Reactome. For each category, the percentage or *p*-value of dysregulated proteins is indicated.

Protein Analysis Through Evolutionary Relationships (PANTHER) is a comprehensive database used to analyze protein family, gene ontology and pathways for proteins with different abundances between adjacent and tumor tissues [22]. PANTHER analysis showed that 146 dysregulated proteins could be categorized into 25 protein classes in which nucleic acid binding proteins comprised the largest group (8.9%) (Figure 3A). According to the Meta-analysis, these proteins were most associated with metabolic (25.5%), and cellular (17.0%) processes, among others (Figure 3B). This study also revealed that molecular functions of these proteins were mostly concerned with catalytic (34.5%) and binding (22.6%) activity (Figure 3C). We performed additional analysis using the Database for Annotation, Visualization and Integrated Discovery (DAVID) in order to further shed light on functional annotation of these dysregulated proteins. DAVID contains an integrated biological knowledgebase and extracts biological meaning from large gene/protein lists at systematically [23]. The results in Table S3 show that 146 differentially express proteins possessed various molecular functions and biological process. Further examination of the group of 65 upregulated proteins, DAVID revealed that their main functions is RNA and protein binding, pointed to biological processes of RNA splicing and processing, as well as metabolic processes (Figure 3D,E and Table S3, Supplementary Material).

To obtain credible signaling pathways where dysregulated proteins may participate, STRING and Reactome were selected to find enriched pathways together with PANTHER and DAVID. STRING is a global scale database that annotates protein interactions and associations at various levels [24]. Reactome is an expert-authored, peer-reviewed knowledgebase of human reactions and pathways that functions as a data mining resource and electronic textbook [25]. Comprehensive analysis by four publicly available pathway tools revealed many enriched pathways take part in GC, for example metabolic pathways, gene expression, the citric acid (TCA) cycle and respiratory electron transport, mitochondrial dysfunction, oxidative phosphorylation, mRNA splicing (Figure 3F and Table S4, Supplementary Material).

**Figure 4:**
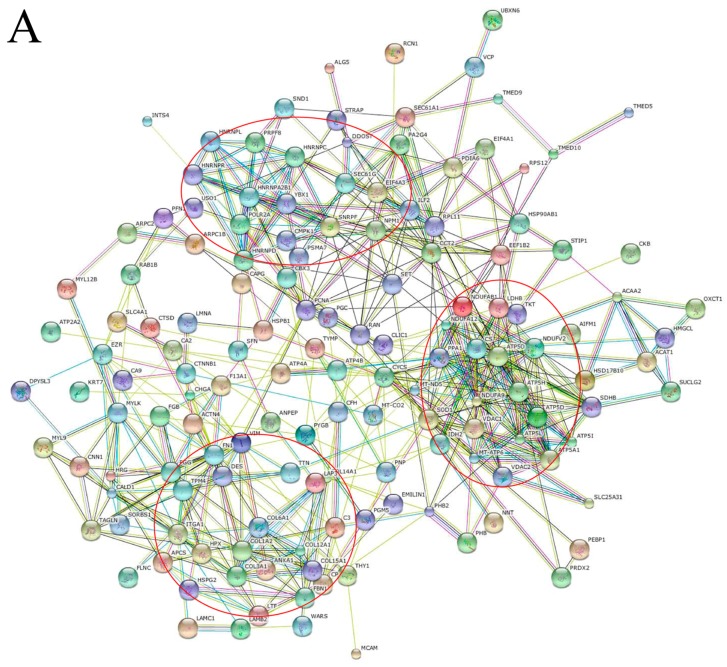

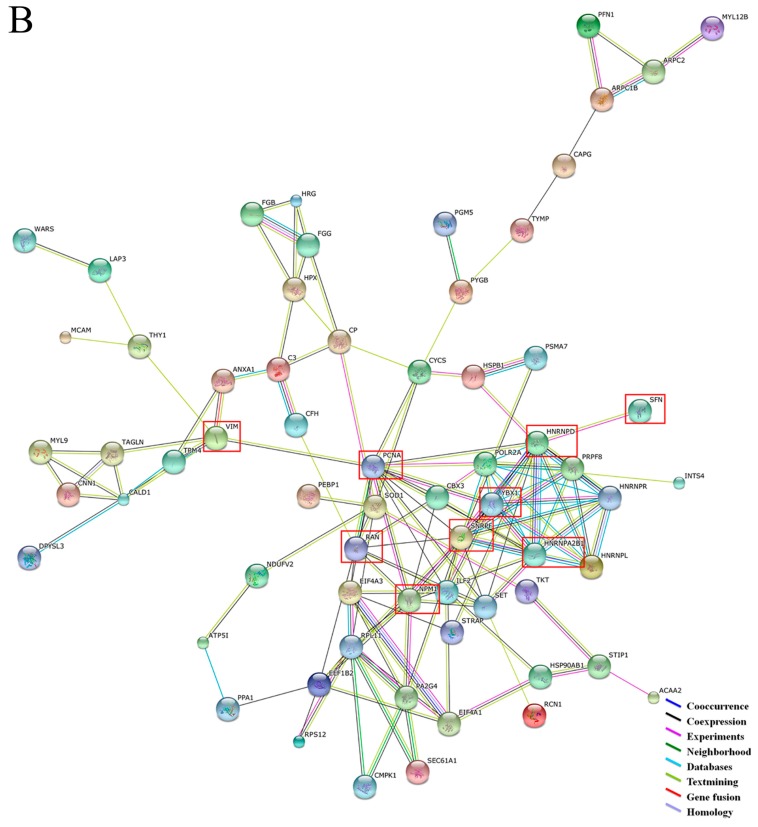
Protein-protein interactions (Evidence Mode) of dysregulated proteins were predicted by STRING. (**A**) Protein-protein interaction network formed with 146 dysregulated proteins. The three possible systematic dynamic clusters were indicated in red circles; (**B**) The network predicted 65 upregulated proteins. Some important proteins dispersed and located in the keynotes were marked with a red box. Different line colors represent the types of evidence for the association.

STRING predicted protein-protein interaction of 146 dysregulated proteins showed that most of them could interact with each other and formed strong networks with three dynamic clusters. The first cluster was mostly populated with nucleic acid binding proteins, such as heterogeneous nuclear ribonucleoproteins (hnRNPs) of hnRNPL, hnRNPR, hnRNPA2B1, hnRNPD and hnRNPC, Y-box binding protein 1 (YBX-1) and nucleophosmin (NPM1). The second cluster was made up of metabolic proteins, for example ATP synthase (ATP5H, ATP5L, ATP5A, ATP5D, ATP5I, MT-ATP6), citrate synthase (CS), NADH dehydrogenase [ubiquinone] 1, superoxide dismutase (SOD). The final cluster identified contained extracellular matrix proteins, such as collagens (COL6A1, COL1A2, COL3A1, COL12A1, COL15A1, COL14A1), fibrillin-1 (FBN1), desmin (DES) and vimentin (VIM) (Figure 4A).We selected 65 upregulated proteins in tumor tissues to further analyze protein-protein interaction as downregulated proteins may not be suitable for potential biomarkers [26]. The results showed that upregulated proteins could also form an interactive network (Figure 4B). In the central network, nucleic acid binding proteins were dispersed and located in the keynotes of network, for example NPM1, YBX-1, hnRNPA2B1, hnRNPD, GTP-binding nuclear protein Ran (RAN), small nuclear ribonucleoprotein F (SNRPF). Additionally, proteins in this cluster extend out to connect with some important functional proteins, such as proliferating cell nuclear antigen (PCNA), VIM, 14-3-3 protein sigma (SFN).

### 2.3. Validation of Dysregulated hnRNPA2B1, hnRNPD, hnRNPL and YBX-1 by Nano-LC-MS/MS, qRT-PCR and Western Blot

**Figure 5:**
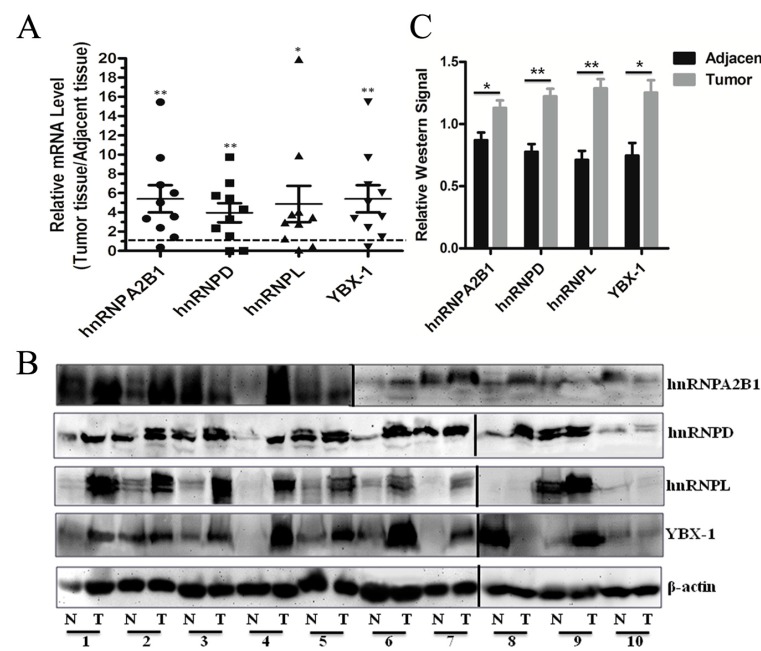
Expression levels of hnRNPs and YBX-1 in GC and adjacent tissues. (**A**) qRT-PCR (*n* = 10) results showing the mRNA expression of hnRNPs and YBX-1. The ratio below the dotted line represented down-expression in GC tissues; otherwise represented up-expression in GC tissues; (**B**) Western blots (*n* = 10) of hnRNPs and YBX-1. N represent adjacent tissue and T represent tumor tissue; (**C**) Grayscale scanning of western blots bands. The ratio was compared to β-actin and statistically analyzed. Significance of differences between GC and adjacent tissues are displayed by ** *p*-value < 0.01 or * *p*-value < 0.05.

Taken together, a number of evidence suggests that activated nucleic acid binding proteins might play a vital role in cell growth, proliferation and metastasis in GC pathogenesis. To verify the activation of these kinds of proteins, Nano-LC-MS/MS, qRT-PCR and western blot were employed to validate the differential expression of hnRNPA2B1, hnRNPD, hnRNPL and YBX-1 in ten pooled or individual GC patients’ tissue samples. By Nano-LC-MS/MS analysis based on relative abundances of Peptide-Spectrum Match (PSMs), significant up-regulations of hnRNPA2B1, hnRNPD, hnRNPL and YBX-1 were confirmed (Tables S5 and S6, Supplementary Materila) [27]. QRT-PCR and western blot both demonstrated a significant up-regulation of three hnRNPs and YBX-1 transcription and translation in the most of the ten GC tissues compared with adjacent tissues, with only 1–2 cases being the exception (Figure 5A–C). These results also demonstrated that qRT-PCR, western blot and a coupled label-free MS approach have consistency in mRNA and protein abundance quantification.

### 2.4. Expression and Distribution Detection of hnRNPA2B1, hnRNPD, hnRNPL and YBX-1 by Immunohistochemistry

**Figure 6:**
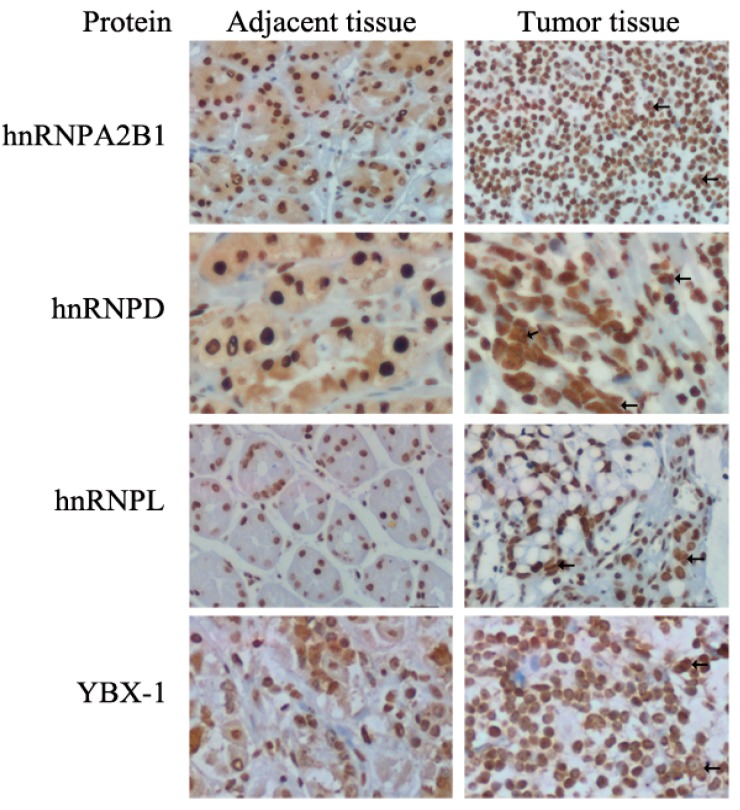
Representative immunohistochemical staining for sectioned formalin fixed GC and adjacent tissues. Specific antibodies of Anti-hnRNPA2B1 (Santa Cruz, TX, USA), Anti-hnRNPD (Proteintech, Chicago, IL, USA), Anti-hnRNPL (Santa Cruz) and Anti-YBX-1 (Santa Cruz) were hybridized respectively. IHC results showed that the morphology of tubular glands disappeared in cancer sections when compared to adjacent tissues. Cancer sections have stronger and higher density nuclei staining, high ratios of nucleus/cytoplasmic area, different shaped nuclei including megakaryocytes and polykaryocytes (arrows). Weak cytoplasmic staining were only seen in hnRNPA2B1, hnRNPD and YBX-1 hybridized normal sections. The magnification is 400×; scale bar: 20 μm.

Proteins highly abundant in tumors might be detectable with better reproducibility by immunohistochemistry (IHC), which can also reveal the distribution status of particular proteins in cells and tissues. With specific antibodies hybridization, IHC results showed a stronger and higher density positive nuclei staining for all three hnRNPs and YBX-1 in GC tissues than in adjacent normal tissues (Figure 6). Cancer cells undergoing cell proliferation have larger and multiple nuclei (arrows shown) compared to normal cells. Furthermore, by observing protein expression and distribution, the slides stained with hnRNPA2B1, hnRNPD and YBX-1 antibodies displayed a weak (1+) to moderate (2+) glandular epithelium cell cytoplasm and nucleus staining in adjacent tissues, whereas there were only strong (3+) nucleus staining in tumor tissues were found. HnRNPL did not appear in cytoplasms. Immunohistochemistry not only confirmed the expression of four upregulated proteins in accordance with qRT-PCR and western blot results, but also revealed nucleo-cytoplasmic shuttling of hnRNPA2B1, hnRNPD and YBX-1.

## 3. Discussion

Since the introduction of label-free proteomics, a broad variety of studies aiming for discovery of biomarkers or drug targets for diseases have been performed [16]. However, this method has been seldom used in GC studies. In 2012, by using shotgun proteomic approach and label-free quantitation analysis, Aquino *et al.* revealed tissue-type proteins were very distinct from each other in control, cancer and resection margin biopsies, only 11, 22, and 29 proteins (*p*-value ≤ 0.05) were quantified in cancer *vs.* control, resection margin *vs.* cancer and resection margin *vs.* control, respectively [28]. Moreover, resection margin biopsies proteins may be related to tumor nourishment and metastasis [28]. In 2013, by using a combinatorial approach of Con-A affinity chromatography, SDS-PAGE, LC/MS/MS and label-free comparative glycoproteomic quantification strategy, Uen *et al.* found 17 differentially expressed glycoproteins with 10 upregulated and 7 downregulated in plasma from GC patients *versus* healthy volunteers [29]. In 2015, by using SDS-PAGE and a coupled label-free MS approach, Qiao *et al.* identified 297, 419, and 265 dysregulated proteins with ≥2 folds in SGC-7901, MGC-803 and HGC-27 cells respectively when compared with GES-1 cells, and provided evidence showing that filamin C is a tumor suppressor, inhibiting cancer cells metastasis [30]. In our study, by using filter-aided sample preparation (FASP) method followed by a coupled label-free MS approach on whole protein extract from surgically resected GC patients’ fresh tumor and matched adjacent tissues, we identified and quantified a higher number of dysregulated proteins. In three independent cases with matched samples, a total of 3639 and 3543 proteins in cancer and adjacent tissues were identified. For better quantification, each of three case samples was performed in triplicates on LC-MS/MS and statistical analysis was carried out. A total of 146 dysregulated proteins with more than twofold differential expression were quantified between tumor and adjacent tissues, 81 of which were downregulated, while the other 65 proteins were upregulated in tumor tissues.

Further analysis indicated that many of these 146 proteins have been aligned with previous studies, such as chloride intracellular channel 1 (CLIC1) [31], SFN [32,33], ATP5A1, carbonic anhydrase 2 (CA2), elongation factor 1-β (EEF1B2), tropomyosin alpha-4 chain (TPM4), PCNA [33], profilin 1 (PFN1), chromobox protein homolog 3 (CBX3) [34], ATP5H [33,34], filamin C [30], calponin-1 (CNN1) [35], heat shock protein β-1(HSPB1) [35]. These proteins have been reported to be associated with poor prognosis, metastasis, aggressiveness, proliferation, migration and invasion, and may be used as diagnostic biomarkers in GC. Meanwhile our data has shown, for the first time, that 22 of 146 dysregulated proteins are related with GC, for example hnRNPD, hnRNPR, ATP5D and EMILIN1. These results not only validate the credibility and effectiveness of our data, but also suggest label-free technique is high throughput approach for identifying proteins with the largest dynamic range and the highest proteome coverage.

Although proteomics approaches focusing on the differences between tumor and adjacent tissues can reveal a number of proteins relevant to tumor, functional annotations of carcinogenesis require bioinformatics and biostatistical tools for analysis, which have become indispensable to handle and to interpret the vast amount of data. In our study, IPA, PANTHER, STRING, DAVID and Reactome were comprehensively used to exploit and explain any possible information related to GC carcinogenesis. By elucidating, 117 and 99 molecules of 146 dysregulated proteins were classified to be related with cancer and gastrointestinal disease development, respectively. Their functions concerning cellular growth and proliferation, nucleic acid metabolism, small molecule biochemistry, cell death and survival, cellular movement were all relative to cancer pathogenesis. Moreover, nucleic acid binding proteins (8.9%) were the most abundant group identified to be predominantly catalytic in nature (34.5%) and greatly involved in metabolic processes (25.5%). Kocevar *et al.* has partially confirmed our hypothesis by identifying 30 different proteins involved in metabolism, development, death, response to stress, cell cycle, cell communication, transport, and cell motility processes in GC [34]. Signal pathway prediction revealed that metabolic pathways, gene expression, oxidative phosphorylation, mitochondrial dysfunction, mRNA splicing were involved in GC carcinogenesis. Protein-protein interaction prediction uncovered an integrated network with three protein clusters. The clusters showed a recruitment of proteins with functional synergy, such as nucleic acid binding associated proteins, metabolizing associated proteins and extracellular matrix associated proteins. It was clear that all these proteins could somehow interact with each other so as to control the cells fate together. In the network consisting of 65 upregulated proteins, a series of hnRNPA2B1, hnRNPD, hnRNPL, hnRNPR together with other nucleic acid binding proteins, such as YBX-1, NPM1, RAN, SNRPF, were gathered in the center of the network, and functionally reacted with some confirmed GC-related proteins such as SFN with carcinogenesis and poor prognosis of GC [32,33], PCNA with abnormal cell proliferation [33], VIM with epithelial-mesenchymal transition in tumor [36] These data remind us that activation of the group of nucleic acid binding proteins might push stomach cells into entering an abnormal proliferation cycle.

To confirm our findings and hypothesis that nucleic acid binding proteins may form a network center and may play a vital role in abnormal cell proliferation during GC development, four upregulated molecules of hnRNPA2B1, hnRNPD, hnRNPL and YBX-1 from the center of the network in Figure 4 were selected to perform further experiments by Nano-LC-MS/MS, qRT-PCR, western blot and immunohistochemistry. The results are consistent with the findings by the coupled label-free MS approach, strongly supporting differential expression of four molecules between GC and adjacent tissues in ten matched case samples.

HnRNPs are a set of primarily nuclear proteins that bind to nascent transcripts with important roles in multiple aspects of nucleic acid metabolism, including the packaging of nascent transcripts, alternative splicing and translational regulation. Their potential roles in tumor development and progression including the inhibition of apoptosis, angiogenesis and cell invasion have been reported [37]. Aberrant expression of hnRNPs in cancers have been detected, for example hnRNPA2B1 in breast, pancreatic and gastric cancer, hnRNPB1 in lung cancer, esophagus cancer, leukemia and lymphoma, hnRNPC1/C2 in lung cancer, hnRNPA2B2 in thyroid cancer and hnRNPM4 in lung cancer [38]. In our study, hnRNPA2B1, hnRNPD, hnRNPL and hnRNPR expression were found to be increased in GC tissues, but hnRNPC expression was decreased. Among these proteins, hnRNPD and hnRNPR were first reported to be relevant with GC. In immunohistochemistry, we observed that hnRNPA2B1 and hnRNPD expression have nucleo-cytoplasmic shuttling phenomenon. Elevated expression of hnRNPA2B1 in GC has been reported [39], which is keeping in line with our results. Translocation of hnRNPA2B1 with *c-myc*, *c-fos*, *p53*, and *Rb* from nucleolus to cytoplasm during tumor cells differentiation [40]. HnRNPA2B1 could act as a novel regulator of oncogenic *K-ras*, modulating PI3K/AKT/mTOR signal pathway in *K-ras*-dependent pancreatic ductal adenocarcinoma cells [41,42]. HnRNPD could enhance the expression of *c-myc*, *c-fos* and *c-jun*, and regulated liver cancer cell proliferation in transgenic mice [43]. Thyroid carcinoma may recruit cytoplasmic hnRNPD to disturb the stability of mRNAs encoding cyclin-dependent kinase inhibitors, leading to uncontrolled growth and progression of tumor cells [44]. As illustrated above, many reports have shown that hnRNPs may be involved in proliferation and metastasis in GC development.

YBX-1 is a multi-functional protein that participates in a wide variety of DNA/RNA-dependent events, and acts as a versatile oncoprotein with an important role in carcinogenesis [45]. In our study, the expression of YBX-1 was upregulated and the distribution of its nucleo-cytoplasmic shuttling was indicated in GC tissues. Previous research reported that YBX-1 was predominantly localized in cytoplasm, particularly in the perinuclear region in benign cells [45]. Environmental stresses, such as adenovirus infection, hyperthermia, oxidative stress, UV irradiation or DNA-damaging drugs, could induce YBX-1 relocation from cytoplasm to nucleus [45]. YBX-1 can activate E2F, PI3K/Akt/mTOR and Ras/Raf/MEK/ERK pathways to promote cancer cell proliferation [46]. YBX-1 expression correlated significantly with lymph node status and perforation and as a potential prognostic biomarker in intestinal-type GC [47]. YBX-1 can promote GC development in both cancer cells and cancer vascular cells [48]. Nuclear YBX-1 expression was significantly associated with Her2 expression, poor prognosis and metastasis in GC patients [49]. In brief, YBX-1 may become a potential biomarker and target molecule in GC therapy.

There are some limitations in our study that need to be addressed. Firstly, the 146 dysregulated proteins were screened from only three GC patients between cancer and adjacent tissues, therefore it is needed to confirm these dysregulated proteins among multiple specimens to avoid heterogeneity and remove artificial differences owning to differential proteins losses during protein or peptide preparation. Second, the sample size still needed to be expanded, particularly for qRT-PCR, western blot and immunohistochemistry analysis. Tissue microarray should be employed to validate the results and candidate molecules.

## 4. Experimental Section

### 4.1. Clinical Tissue Samples

Ten cases of GC and adjacent normal tissues were collected from GC patients who underwent gastric resection at the Department of Digestive Diseases, Xijing Hospital Affiliated to Fourth Military Medical University between November 2012 and January 2013. GC patients were male, ages from 40 to 70 years old, with primary and low to moderate differentiated gastric adenocarcinoma. Radiotherapy, chemotherapy, and immunotherapy were not performed before surgery. Cancer tissues were taken from the core area of tumor, avoiding inclusion of necrotic and adjacent non-cancerous tissues. Adjacent tissues were prepared from non-cancerous regions at least 5 cm apart from the core area of tumor. All samples were verified by two pathologists after surgery. Clinical pathologic characteristics are described in Table S7 (Supplementary Material). Tissue samples were obtained with informed patient consent and approved by the Medical Ethics and Human Clinical Trial Committee of Xijing Hospital (Approval No.: XJYYLL-2015694 and Approval Date: 4 March 2015).

### 4.2. Protein Preparation

Whole proteins were extracted from tissues as previously reported [17]. Matched GC and adjacent fresh tissues were excised immediately following gastrectomy, cut into small blocks, and rinsed with ice-cold PBS. About 100 mg GC or adjacent tissues were homogenized in RIPA lysis buffer (Pierce, Thermo Scientific, Waltham, MA, USA) containing protease inhibitor cocktail (Roche, Basel, Switzerland). Tissue lysates were centrifuged at 15,000× *g* for 20 min at 4 °C, and supernatants were collected. After protein concentration was determined by BCA (bicinchoninic acid) protein assay kit (Pierce, Thermo Scientific, Germany), protein aliquots were stored at −80 °C.

### 4.3. In-Solution Tryptic Digestion

Proteins from three pairs of GC and adjacent tissues were processed in-solution digestion respectively as previously described [17,26]. The samples were dialyzed with ammonium bicarbonate, reduced with DL-Dithiothreitol (DTT, Sigma-Aldrich, St. Louis, MO, USA), and alkylated with iodoacetamide (IAA, Sigma-Aldrich). Trypsin (Sigma-Aldrich) digestion was performed at 37 °C for 24 h, and C18 spin columns (Millipore, Waltham, MA, USA) were used to purify the peptides. Peptides were dried in SpeedVac and stored at −80 °C until analysis.

### 4.4. SDS-PAGE and in-Gel Tryptic Digestion

About 100 μg protein were run on 12% sodium dodecyl sulfate polyacrylamide gel electrophoresis (SDS-PAGE). Proteins from 10 samples of GC or adjacent tissue lysates were pooled to avoid heterogeneity. After coomassie blue staining, the protein bands were cut into 10 equal shares from the gel, processed in-gel digestion with trypsin using the standard protocol [50]. After trypsin digestion, Ziptip C_18_ micropipette tips (Millipore) were used to purify the peptides prior to adding 0.1% formic acid for Nano-LC-MS/MS analysis.

### 4.5. Nano-LC-MS/MS Analysis

The method was performed as previously described [26,50]. In brief, peptides of three individually or ten pooled tryptic samples were fractionated by high pressure liquid chromatography (HPLC, Thermo EASY-nLC System, Waltham, MA, USA) respectively: buffer A (0.1% (*v*/*v*) formic acid in Milli-Q water) and buffer B (0.1% formic acid in 100% acetonitrile). Peptides were eluted from the column at a constant flow rate of 300 nL·min^−1^ with a linear gradient of buffer B from 5% to 35% over 120 min. Then following full scan with LTQ Velos Pro tandem mass spectrometer (Thermo Fisher Scientific, Waltham, MA, USA): LTQ MS/MS depended scans with collision-induced dissociation (CID) mode. Each of the three tryptic samples must be loaded three times with the same methods. MS/MS data were analyzed with Proteome Discoverer 1.4 (Mascot and SEQUEST, Waltham, MA, USA) according to manufacturer’s instruction and searched against human uniprot protein database (UniProtKB [51] 3 November 2014, 140,992 sequences) for protein identification. Peptide mass tolerance was set to 0.8 Da, fragment mass tolerance was set to 10 ppm and a maximum of two missed cleavages was followed. Variable modification was oxidation of methionine, static modification was carbamidomethylation of cysteine. Protein identification was considered valid if at least one peptide and the *p*-value <0.05, the proteins not satisfying these defined criteria were rejected, the threshold for accepting MS/MS spectra was false discovery rate (FDR) 0.05.

### 4.6. Label-Free Quantification

Label-free quantification of three matched samples was acquired and analyzed in Progenesis LC-MS software (version 4.1, Milford, MA, USA) as previously described [50,52]. Mass spectrometer raw-files were transformed to .mzxml with ReAdw-program and imported into Progenesis LC-MS software. The ion intensity maps of all six runs were examined for defects. One sample was set as the reference, data processing was aligned, and peptide ions with charge state of +1 or >4 and difference ratio of proteins (adjacent/cancer) are <2.0-fold or >0.5-fold were excluded. For quantification, the unique peptides validated by MS (*p*-value < 0.05) were chosen and calculated by summing the abundances of all peptides allocated to a specific protein. The software calculates ANOVA and q-values, which were used to deduce differentiating peptides.

### 4.7. Bioinformatics Analysis

Limited selections were used to screen label-free quantitative data before our results were necessary for differential analysis: proteins with the same peptides found in two or three patients were considered; retrieve the credibility is ≥95%, false positive is <5% in the database; difference ratio of proteins is ≥2.0-fold and *p*-value is ≤0.05; identified proteins must be redundancy by all artificial. Dysregulated proteins were subjected to bioinformatics analysis tools for enrichment categories of functional annotation, networks and diseases-related proteins, for example PANTHER 9.0 [53], IPA [54], STRING (version 9.1) [55], Reactome [56] and DAVID (Bioinformatics Resources 6.7) [57].

### 4.8. Validation of Dysregulated Proteins by qRT-PCR, Western Blot and Immunohistochemistry

Tissues were ground with mortar and total RNA was extracted with RNAfast 1000 (Pioneer biotechnology, Xi’an, China), and reverse transcribed with PrimeScript™ RT Reagent kit with gDNA Eraser (TaKaRa, Tokyo, Japan) according to the respective manufacturers’ instructions. Diluted aliquots of reverse transcribed cDNAs were used as templates in qRT-PCR containing SYBR Green PCR Master Mix (TaKaRa) with Applied Biosystems 7500 Fast Real-Time PCR system (Life technologies, Waltham, MA, USA). Triplicate reactions were carried out for each sample to ensure reproducibility. Gene expression was quantified using the comparative cycle threshold (*C*_t_) method. Primers sets are listed in Table S7 (Supplementary Material).

Proteins from adjacent and tumor tissues were separated on SDS-polyacrylamide gels were transferred to PVDF membranes by electro blotting. Membranes were washed and blocked, then were incubated with the specific primary antibodies (Table S7, Supplementary Material) at 4 °C overnight. Membranes were washed with PBST and incubated with the respective HRP-conjugated secondary antibody. Signals were visualized with the WesternBright™ Sirius Highest sensitivity chemiluminescent HRP Substrate (Advansta, Menlo Park, CA, USA) and intensities recorded with a ChemiDoc-It 510 Imager (UVP, Upland, CA, USA). Band intensities were quantified using the Image J and normalized to total protein on the membrane.

Immunohistochemistry analysis was performed with GC and adjacent tissues fixed in 10% formalin, embedded in paraffin, and sectioned at 3–4 microns. Tissue sections were deparaffinized, hydrated, subjected to thermal treatment in Tris–EDTA in a pressure cooker at boiling for 20 min for antigen retrieval, exposed to endogenous peroxidase blocking and incubated with primary antibodies (Table S7, Supplementary Material) for 2 h at room temperature. The reaction was visualized with 3, 3′-diaminobenzidine tetra hydrochloride (DAB, Zhongshan, China) as chromogen. Finally, sections were counterstained with hematoxylin, dehydrated, mounted, and tissue slides were evaluated under a microscope (Olympus IX71, Olympus Corporation, Tokyo, Japan). For evaluating protein expression, the intensity of staining was scored as negative, weak, moderate, or strong (score 0, 1, 2, or 3, respectively) [26].

### 4.9. Data Analysis

SPSS 19.0 software (SPSS Inc., Armonk, NY, USA) was used for statistical analysis. *p*-value < 0.05 was considered statistically significant. Statistical analyses between two groups were performed using a two-tailed Student’s *t*-test.

## 5. Conclusions

In summary, we confidently identified 146 dysregulated proteins including 22 proteins first reported on paired GC and adjacent tissues by a coupled label-free MS approach. Additionally, we noted the majority of dysregulated proteins were involved in cancers and gastrointestinal disease. Moreover, four possible key carcinogenesis molecules of hnRNPA2B1, hnRNPD, hnRNPL and YBX-1 were validated by Nano-LC-MS/MS, qRT-PCR, western blot and immunohistochemistry. They were located in a predicted interaction network keynotes and their nucleo-cytoplasmic shuttling may play an important role in gastric carcinogenesis. Although further studies are needed to validate their functional roles and molecular mechanism, these findings provide an overview and deeper understanding about GC-related molecular changes, and provide a group of potential diagnostic/prognostic biomarkers and therapeutic molecular targets for clinical intervention of GC.

## Supplementary Materials

Supplementary materials can be found at http://www.mdpi.com/1422-0067/17/1/69/s1.
